# Recent trends in dialysis initiation in Japan: a region‑specific descriptive analysis using Hokkaido as an example

**DOI:** 10.1007/s10157-026-02831-y

**Published:** 2026-02-26

**Authors:** Hirofumi Sakuma, Megumi Matsumoto, Yusuke Kanno, Saeko Miura, Reina Suetsugu-Ishizawa, Nozomi Hayashi, Motoki Matsuki, Atsushi Wada, Naoki Nakagawa

**Affiliations:** 1 Division of Cardiology and Nephrology, Department of Internal Medicine, Asahikawa Medical University, Midorigaoka-Higashi 2-1-1-1, Asahikawa, Japan; 2 Department of Nephrology, Kitasaito Hospital, Asahikawa, Japan

**Keywords:** Diabetic kidney disease, Dialysis initiation, Japanese registry, Nephrosclerosis, Regional differences

## Abstract

**Background:**

The increasing number of dialysis patients presents a significant public health challenge in Japan. While the number of patients initiating dialysis due to chronic glomerulonephritis (CGN) or diabetic kidney disease (DKD) has decreased, the incidence of dialysis initiation attributed to nephrosclerosis has gradually increased. To investigate regional differences, Hokkaido, Japan, was selected as one regional example in this study.

**Methods:**

Data were extracted from a web-based national database for patients aged ≥ 40 years who initiated dialysis between 2012 and 2021. Patients were categorized according to sex, age, and underlying disease. Dialysis initiation rates were calculated as the number of new dialysis patients divided by the corresponding population. To assess temporal changes, the 2021/2016 ratio was calculated by dividing the initiation rate in 2021 by that in 2016 for each prefecture.

**Results:**

In Hokkaido, the dialysis initiation rates for DKD declined across all age groups, whereas the national rate increased in men aged ≥ 80 years. For nephrosclerosis, the initiation rates rose among older adults both nationally and in Hokkaido, although the increase in Hokkaido was more gradual. CGN-related rates decreased consistently across all age groups, both nationally and in Hokkaido. Prefectures with 2021/2016 ratio of ≥ 1 were more frequently observed among patients with nephrosclerosis than among those with CGN or DKD, especially among older populations.

**Conclusions:**

Dialysis initiation rates in Hokkaido decreased in most subgroups compared with national trends. Given the rising incidence of nephrosclerosis among older adults, targeted interventions at the prefectural level are urgently warranted.

**Supplementary Information:**

The online version contains supplementary material available at 10.1007/s10157-026-02831-y.

## Introduction

The growing number of Japanese patients undergoing dialysis, which is approximately 350,000 as of 2019 [1] poses a significant challenge to the national Japanese healthcare system. The median age of these patients is 68.4 years, with approximately 70% aged over 65 years [1, 2], reflecting a clear shift toward an aging dialysis population. In addition to the demographic burden, the economic impact is considerable. The annual cost of dialysis treatment is estimated at approximately 5 million yen per patient, accounting for approximately 4% of Japan’s total healthcare expenditure [3]. In response to the rising number of patients undergoing dialysis, the aging population, and the associated healthcare costs, the Japanese government has implemented nationwide strategies for reducing the incidence of dialysis initiation [4]. Therefore, trends in dialysis initiation have become essential as a key performance indicator for evaluating the effectiveness of these policy measures.

The most common underlying disease is diabetic kidney disease (DKD), followed by chronic glomerulonephritis (CGN) and nephrosclerosis [1, 5]. Notably, the number of patients initiating dialysis due to nephrosclerosis has been increasing in Japan; however, the incidence of dialysis attributed to DKD has remained stable, whereas that associated with CGN has gradually declined over the past 15 years [6]. Although previous studies have described national trends in dialysis initiation by underlying disease [6, 7], nationwide statistics may mask substantial regional heterogeneity, as opposing trends across prefectures are averaged out, potentially giving a misleading impression of uniformity. This raises concerns that uniform CKD countermeasures may not adequately address region‑specific needs. In this context, we hypothesized that regional dialysis initiation patterns would diverge from national trends and that identifying such discrepancies is essential for developing locally adapted CKD strategies. Thus, this study investigated dialysis initiation trends in Hokkaido, as one regional example, and compared them with national data. Hokkaido was selected because its demographic, geographic, and healthcare characteristics—including a marked uneven distribution of physicians such as nephrologists—differ from those of many other prefectures, and because it represents the authors’ own residential and clinical practice area where CKD countermeasures are directly implemented. To facilitate the exploration of region-specific dynamics and demonstrate how prefectural-level data can be used to monitor and interpret local trends, data were obtained from the Web-based Analysis of Dialysis Data Archives (WADDA) database [8].

## Methods

### Study design and data source

This observational study used retrospective data from the Renal Data Registry (JRDR) of the Japanese Society for Dialysis Therapy (JSDT) [7]. The JSDT conducts an annual questionnaire survey of all dialysis facilities in Japan, with a response rate of > 98% [1]. The survey collects information on epidemiologic characteristics, treatment modalities, and outcomes related to dialysis therapy. In 2006, the WADDA system was introduced as an automated data transfer platform from the JRDR, allowing members of the JSDT to access patient information [7, 9].

This study included patients aged ≥ 40 years of age who initiated dialysis. Dialysis modalities included hemodialysis, hemodialysis filtration, peritoneal dialysis, and their combinations. Using the WADDA system, data on the number of patients who initiated dialysis between 2012 and 2021, including information on sex, age, underlying disease, and prefecture, were extracted. The population data for each group according to sex, age, and prefecture between 2012 and 2021 were obtained from the national census. The sex-, age-, underlying disease-, and region-specific dialysis initiation rates were calculated by dividing the number of patients initiating dialysis by the corresponding population size in each subgroup.

### Patient characteristics

Clinical data of all patients who initiated dialysis in 2021 in Hokkaido and throughout Japan were extracted using the WADDA system. These data included key variables such as age, dialysis modality, prevalence of comorbidities, body weight, body mass index (BMI), pre-dialysis systolic and diastolic blood pressure, pulse rate, use of antihypertensive drugs, and laboratory data including hemoglobin, total cholesterol, high-density lipoprotein (HDL) cholesterol, non-HDL cholesterol, albumin, calcium, phosphate, intact parathyroid hormone, and C-reactive protein levels. Patients with missing data were excluded from the analysis. Using the 2021 dataset, the clinical characteristics of patients initiating dialysis in Hokkaido were summarized and compared with those of patients nationwide.

### Calculation of the 2021/2016 ratios of dialysis initiation rates by sex, age, underlying diseases, and prefecture

The ratios of dialysis initiation rates in 2021 to those in 2016 (hereafter referred to as “2021/2016 ratios”) were calculated by dividing the dialysis initiation rates in 2021 by those in 2016 according to sex, age, underlying disease, and prefecture. A 2021/2016 ratio ≥ 1 indicated an increase in the number of patients initiating dialysis, while a 2021/2016 ratio of < 1 indicated a decrease.

### Statistical analysis

Data on clinical characteristics were expressed as the means with standard deviations or counts with percentages, as appropriate. Comparisons between subgroups were conducted using the chi-square test for categorical variables and the unpaired t-test for continuous variables. To evaluate temporal trends in dialysis initiation rates, simple linear regression analysis was performed, with calendar year as the independent variable. Results were expressed as a slope coefficient (β) with the corresponding 95% confidence intervals. Additionally, differences in regression slopes between sex and regional groups were assessed using an F-test. A two-sided *p*-value of < 0.05 was considered statistically significant. Statistical analysis was performed using GraphPad Prism 10 for Windows, version 10.2.3 (GraphPad Software, San Diego, CA, USA), and IBM SPSS Statistics for Windows, version 26.0 (IBM Corp., Armonk, New York, USA). Graphs were generated using GraphPad Prism 10 for Windows, version 10.2.3.

## Results

### Demographic characteristics of new patients initiating dialysis in all of Japan and the Hokkaido region

Table 1 shows the demographic characteristics of new patients who initiated dialysis in 2021 in all of Japan and the Hokkaido region. In both populations, the number of male patients was approximately twice that of female patients. Patients nationwide were older than those in Hokkaido in both sexes. No significant differences were observed between the two regions in terms of dialysis modality or the prevalence of comorbidities. However, patients in Hokkaido had significantly higher body weight and BMI compared with those in all of Japan. Physiological measurements and laboratory findings did not differ significantly between the two regions.

**Table 1:** Demographic clinical characteristics of the whole study population

	Women			Men		
	All of Japan	Hokkaido	*p*	All of Japan	Hokkaido	*p*
Number of participants	11,640	527		26,321	1123	
Age (y)	72.7 (13.7)	71.1 (13.7)	*p* < *0.05*	70.4 (13.2)	69.5 (13.3)	*p* < *0.05*
Dialysis modality (%)						
Hemodialysis	65.9	64.3	*N.S*	63.4	61.2	*N.S*
Hemodialysis filtration	27.5	28.4	*N.S*	30.6	32.7	*N.S*
Peritoneal dialysis	6.5	7.3	*N.S*	6.0	6.1	*N.S*
Other	0.0	0.0	*N.S*	0.0	0.0	*N.S*
Comorbidities (%)						
Diabetes mellitus	50.1	52.0	*N.S*	60.4	62.1	*N.S*
Ischemic heart disease	14.3	14.3	*N.S*	21.2	21.7	*N.S*
Cerebral hemorrhage	3.7	2.7	*N.S*	4.1	3.8	*N.S*
Cerebral infarction	10.7	11.9	*N.S*	13.9	15.2	*N.S*
Amputation	1.2	0.8	*N.S*	1.9	1.6	*N.S*
Anthropometric measurements (pre-dialysis)						
Body weight (kg)	51.4 (12.2) [8,930]	53.1 (13.0) [437]	*p* < *0.01*	63.8 (14.0) [20,527]	65.8 (14.3) [918]	*p* < *0.01*
Body mass index (kg/m^2^)	21.5 (4.1) [7,565]	22.1 (4.4) [392]	*p* < *0.05*	22.5 (3.7) [17,290]	23.2 (3.9) [771]	*p* < *0.01*
Physiological measurements (pre-dialysis)						
Systolic blood pressure (mmHg)	148.7 (24.4) [8,966]	149.2 (24.4) [438]	*N.S*	149.3 (23.5) [20,577]	149.2 (23.6) [907]	*N.S*
Diastolic blood pressure (mmHg)	75.5 (14.2) [8,965]	75.4 (14.1) [438]	*N.S*	77.8 (14.3) [20,564]	77.1 (14.1) [907]	*N.S*
Pulse rate (bpm)	74.8 (13.2) [8,791]	75.9 (12.9) [430]	*N.S*	73.7 (13.3) [20,178]	72.3 (12.9) [882]	*p* < *0.01*
Laboratory findings (pre-dialysis)						
Hemoglobin (g/dL)	10.7 (1.5) [8,933]	10.7 (1.5) [435]	*N.S*	10.8 (1.5) [20,482]	10.8 (1.6) [900]	*N.S*
Total cholesterol (mg/dL)	165.9 (40.5) [7,149]	167.3 (42.1) [350]	*N.S*	148.2 (35.6) [16,413]	148.5 (38.3) [737]	*N.S*
HDL cholesterol (mg/dL)	53.5 (18.0) [6,959]	53.9 (17.5) [350]	*N.S*	47.2 (16.2) [16,194]	45.4 (15.1) [760]	*p* < *0.01*
Non-HDL cholesterol (mg/dL)	112.5 (36.7) [6,007]	116.7 (36.8) [293]	*N.S*	101.1 (33.6) [13,937]	103.3 (35.6) [653]	*N.S*
Albumin (g/dL)	3.4 (0.5) [8,933]	3.4 (0.5) [434]	*N.S*	3.4 (0.5) [20,454]	3.4 (0.5) [897]	*N.S*
Serum calcium (mg/dL)	9.1 (0.8) [8,873]	9.1 (0.8) [433]	*N.S*	8.9 (0.7) [20,308]	8.9 (0.6) [897]	*N.S*
Serum phosphate (mg/dL)	5.1 (1.5) [8,942]	5.1 (1.4) [432]	*N.S*	5.1 (1.5) [20,570]	5.2 (1.6) [901]	*N.S*
Serum intact-PTH (mg/dL)	209.2 (205.4) [7,480]	207.2 (224.3) [370]	*N.S*	193.8 (159.0) [17,066]	178.7 (182.1) [773]	*p* < *0.05*
C-reactive protein (mg/dL)	0.7 (2.0) [7,892]	0.7 (2.1) [377]	*N.S*	0.8 (2.0) [17,976]	0.9 (2.5) [791]	*N.S*
Medication						
Anti-hypertensive drug (%)	70.6	71.7	*N.S*	72.3	72.4	*N.S*

Values are expressed as the mean (standard deviation) and [actual number in each column]. HDL, high-density lipoprotein; PTH, parathyroid hormone

### Ten-year trends in dialysis initiation rates by sex and age in all of Japan and the Hokkaido region

**Fig. 1:**
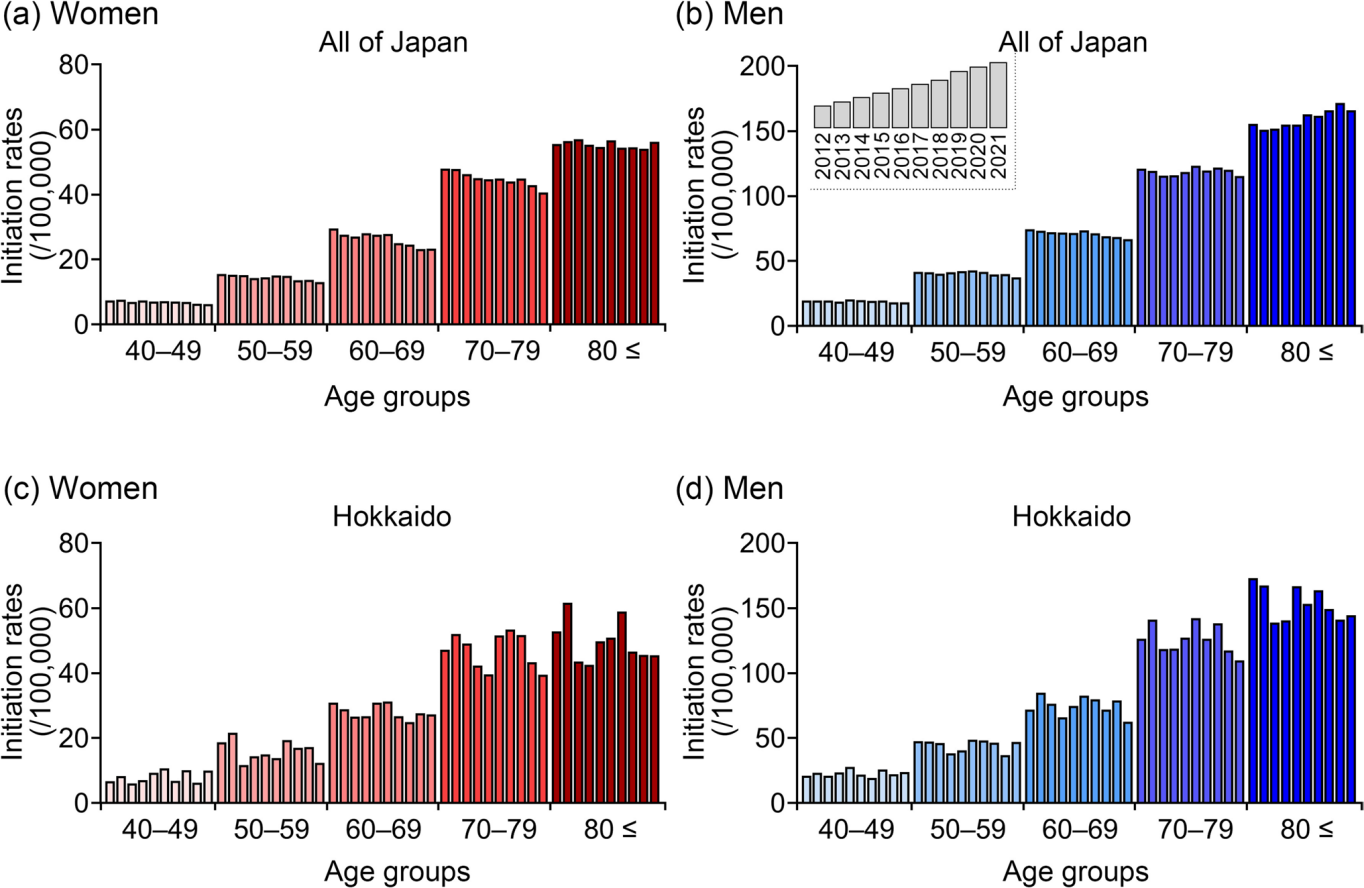
Trends in age-specific dialysis initiation rates between 2012 and 2021 among patients aged ≥ 40 years, stratified by 10-year age groups in Japan (**a**–**b**) and Hokkaido (**c**–**d**). Red bars represent female patients (**a**, **c**), whereas blue bars represent male patients (**b**, **d**). Each panel represent patients stratified by 10-year age group. Dialysis initiation rates were calculated by dividing the number of patients initiating dialysis by the total population in the corresponding population

Figure 1 shows the dialysis initiation rates stratified by 10-year age groups in all of Japan and the Hokkaido region. In both regions, a gradual decline over time was observed in most age groups among women (Fig. 1a and c). Notably, the decrease in dialysis initiation rates among women aged ≥ 80 years in Hokkaido was more pronounced compared with the corresponding national trend (Supplementary Fig. S1a and Table S1). In addition, among men aged ≥ 80 years in Hokkaido, a gradual decline in initiation rates was also observed, whereas a significant increase over time was noted in the same age group at the national level (Fig. 1b and d, Supplementary Fig. S1b, and Supplementary Table S1). Furthermore, at the national level, the trend in dialysis initiation among men aged ≥ 80 years significantly increased compared with that of women, both in the overall population and among patients with DKD or nephrosclerosis. By contrast, no such sex-specific differences were observed in Hokkaido (Supplementary Fig. S2). Across both regions, patients aged ≥ 80 years consistently exhibited the highest dialysis initiation rates among all age groups (Fig. 1a–d).

### Regional variations in 2021/2016 ratios by sex, age, and underlying diseases

**Fig. 2:**
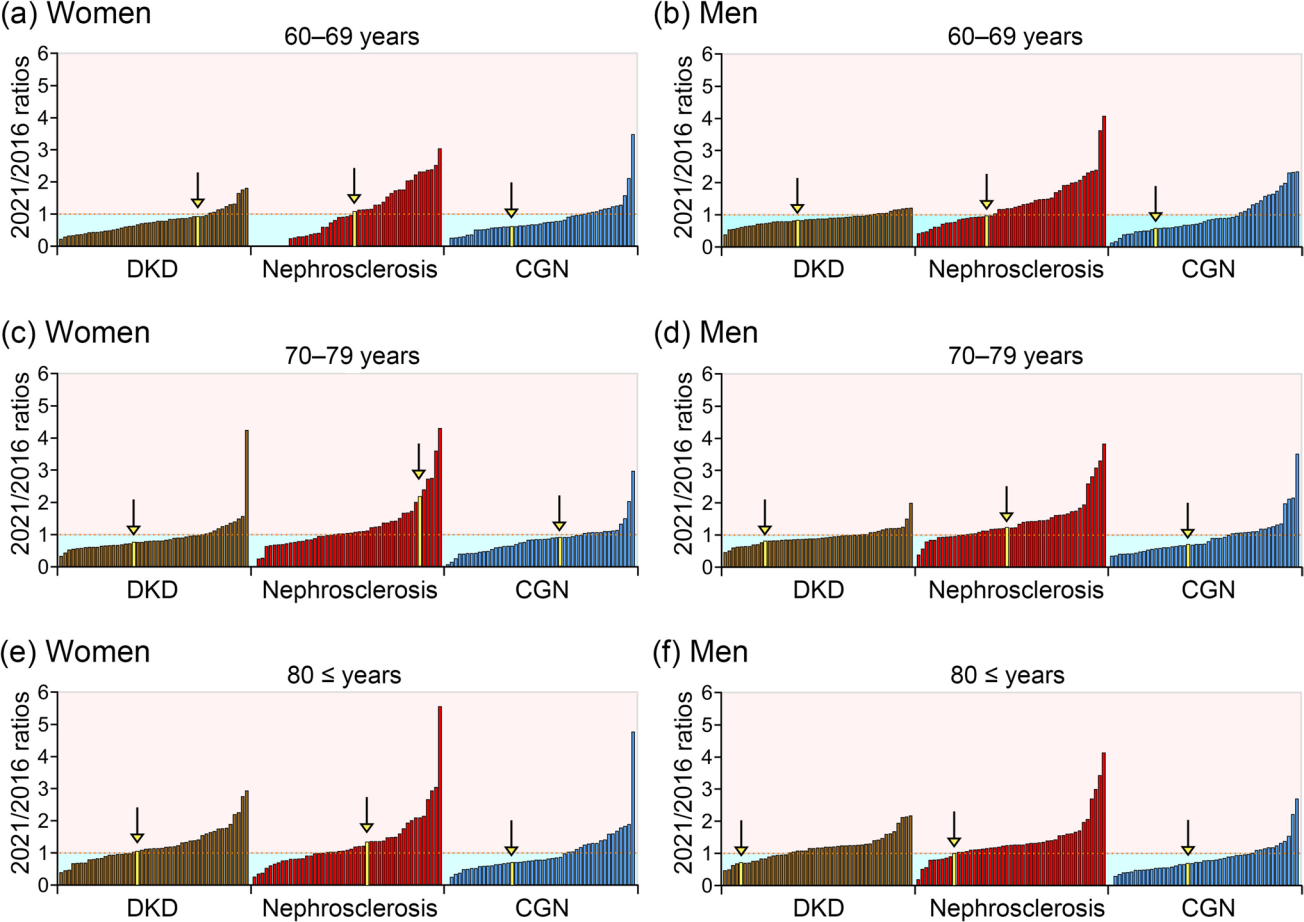
Ratios of dialysis initiation rates in 2021 compared to 2016, stratified by sex, 10-year age groups, underlying disease, and prefectures. The left panels **a**, **c**, **e** represent data for female patients, and the right panels **b**, **d**, **f** represent data for male patients in their 60 s, 70 s, and 80 s and older. Brown bars indicate the 2021/2016 ratios in patients with DKD, the red bars indicate the 2021/2016 ratios in patients with nephrosclerosis, and the blue bars indicate the 2021/2016 ratios in patients with CGN. Each bar represents the 2021/2016 ratios of each prefecture. The horizontal dashed lines indicate a 2021/2016 ratio of 1. Yellow bars and bottom arrows indicate the data in Hokkaido. DKD, diabetic kidney disease; CGN, chronic glomerular nephritis

Figure 2 presents the 2021/2016 dialysis initiation rate according to sex, age, and underlying diseases across the 47 prefectures. Overall, the proportion of prefectures with ratios ≥ 1 was generally higher among older patients compared with younger patients. Similarly, this proportion was also higher in patients with nephrosclerosis than in those with DKD or CGN. Notably, substantial variation was observed among prefectures within the same categorical groups. In particular, the majority of prefectures showed ratios < 1 among patients in their 60 s or 70 s with DKD or CGN, whereas ratios ≥ 1 were more commonly observed in patients with nephrosclerosis across most subgroups. The position of Hokkaido within each categorical group is highlighted using yellow bars and downward-pointing yellow arrows. In Hokkaido, the 2021/2016 ratios were < 1 in most subgroups, except for patients with nephrosclerosis, including women of all ages and men in their 70 s (Fig. 2a–f).

### Ten-year trends in dialysis initiation rates by sex, age, and underlying diseases in all of Japan and the Hokkaido region

**Fig. 3:**
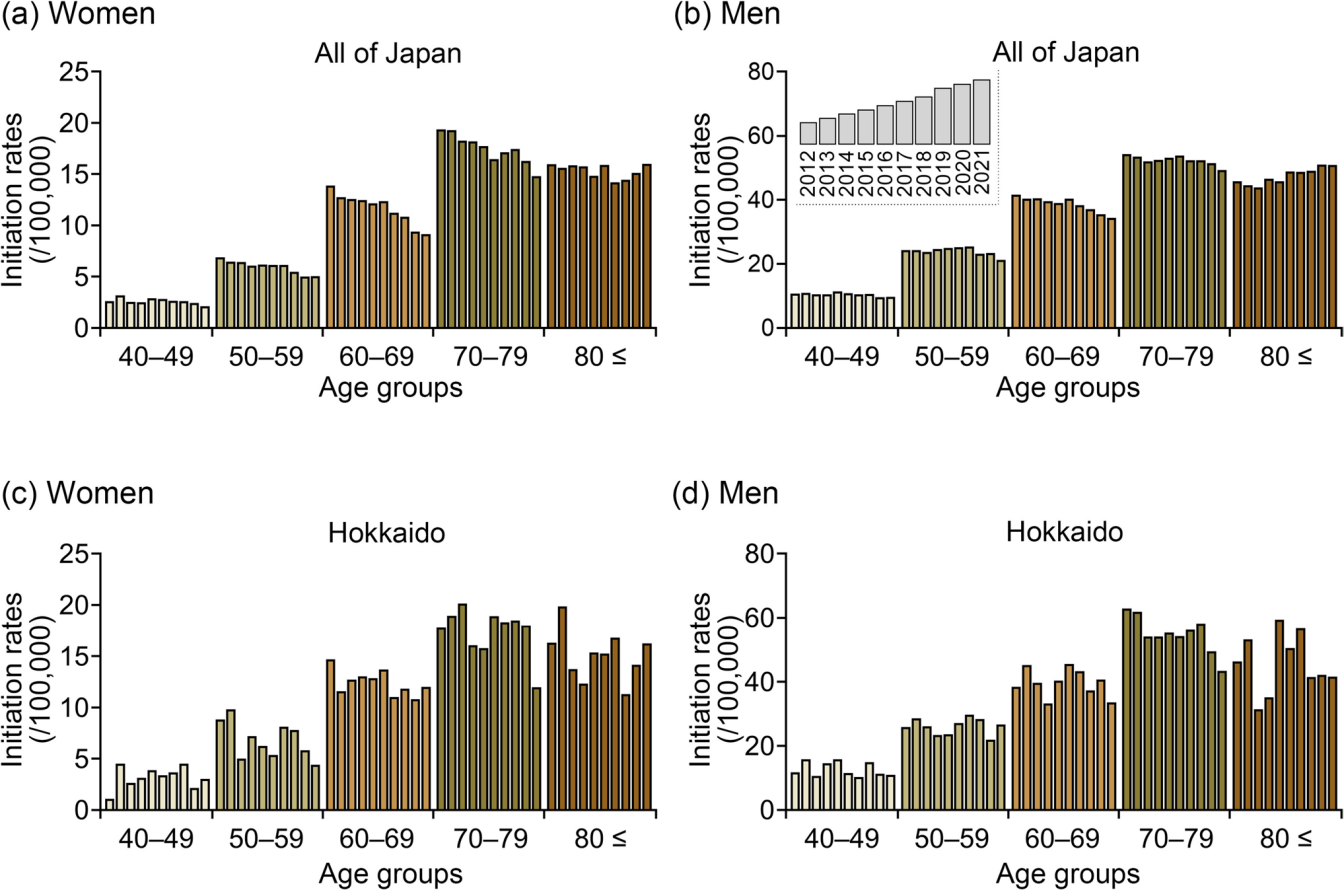
Trends in age-specific dialysis initiation rates between 2012 and 2021 among female (**a**, **c**) and male (**b**, **d**) patients with diabetic kidney disease (DKD) in all of Japan and the Hokkaido region. The age-specific initiation rates were calculated by dividing the number of patients who initiated dialysis by the population in the corresponding age and sex group. The brown bars indicate patients initiating dialysis with DKD (**a**–**b**, in Japan; **c**–**d**, in Hokkaido)

**Fig. 4:**
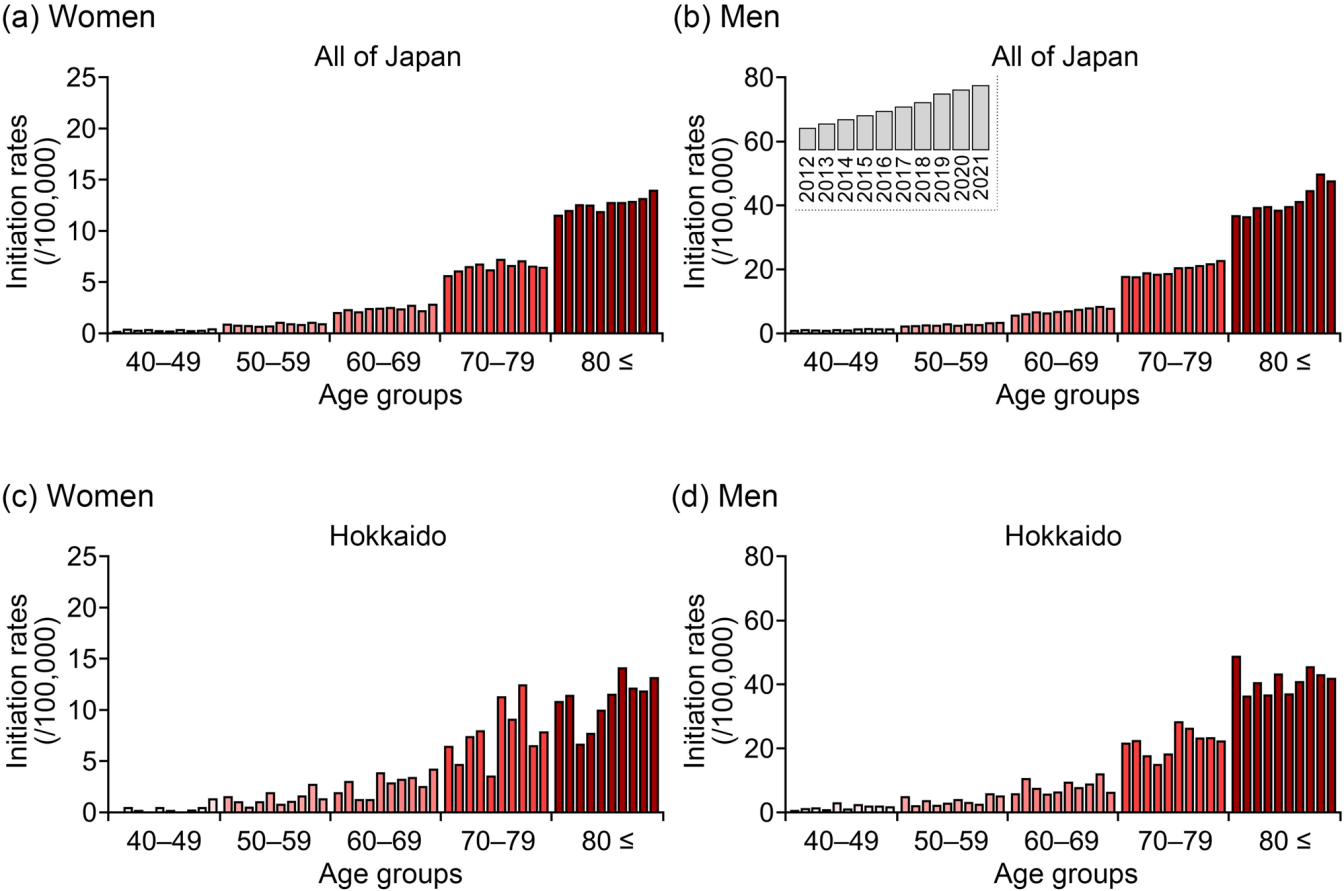
Trends in age-specific dialysis initiation rates between 2012 and 2021 among female (**a**, **c**) and male (**b**, **d**) patients with nephrosclerosis in all of Japan and the Hokkaido region. The age-specific initiation rates were calculated by dividing the number of patients who initiated dialysis by the total number of individuals in the corresponding age and sex group. The red bars indicate patients initiating dialysis due to nephrosclerosis (**a**–**b**, in Japan; **c**–**d**, in Hokkaido)

**Fig. 5:**
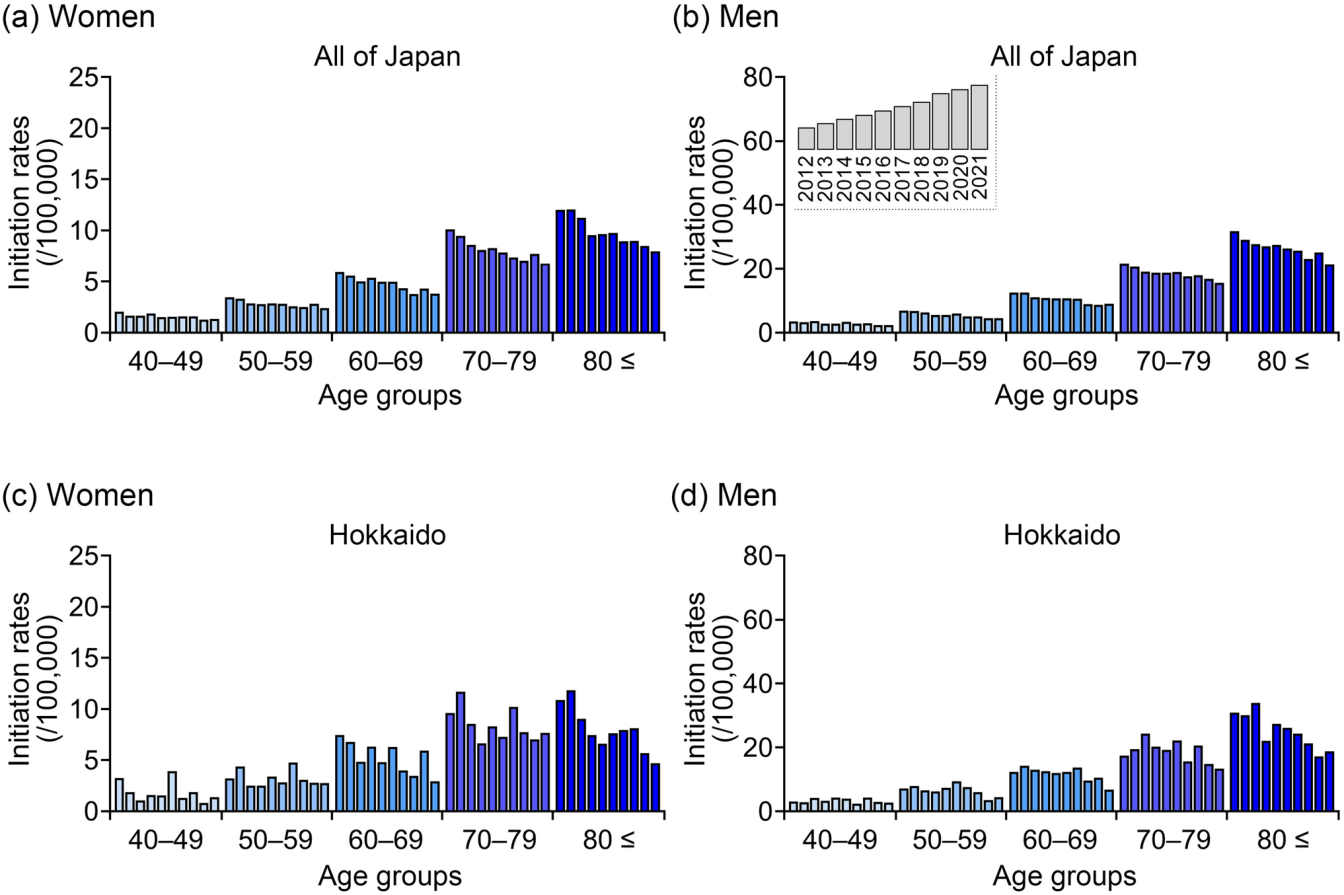
Trends in age-specific dialysis initiation rates between 2012 and 2021 among female (**a**, **c**) and male (**b**, **d**) patients with chronic glomerular nephritis (CGN) in all of Japan and the Hokkaido region. The age-specific initiation rates were calculated by dividing the number of patients who initiated dialysis by the total number of individuals in the corresponding age and sex group. The blue bars represent patients initiating dialysis due to CGN (**a**–**b**, in Japan; **c**–**d**, in Hokkaido)

Figure 3 shows the 10-year trends in dialysis initiation rates among patients with DKD by sex and age group, in all of Japan and the Hokkaido region. Among male patients aged ≥ 80 years with DKD, a significant upward trend was observed nationwide, whereas a gradual decline was noted in Hokkaido (Supplementary Fig. S1 and Table S2). Regional differences in trends among patients with DKD were not significant in most age groups, except for male patients aged 70–79 years (Supplementary Table S2). By contrast, the trends among female patients with nephrosclerosis were similar between the two regions. Among male patients with nephrosclerosis, dialysis initiation rates remained stable in those aged ≥ 70 years in Hokkaido, whereas a significant increase was observed in the corresponding age groups the national level (Fig. 4 and Supplementary Table S3). Particularly, regional differences in trends were particularly evident among male patients aged ≥ 80 years with nephrosclerosis, for whom the differences between Hokkaido and the national data were significant (Supplementary Fig. S1 and Table S3). By contrast, among patients with CGN, the dialysis initiation rates gradually decreased across all age groups in both regions (Fig. 5), with no significant regional differences observed (Supplementary Table S4).

## Discussion

Using the WADDA system database, recent trends in dialysis initiation rates in Japan were comprehensively summarized by sex, age, underlying diseases, and prefecture, to provide foundational descriptive data for the development of CKD countermeasures. Rather than aiming to generalize findings to all prefectures, we selected Hokkaido to conduct a descriptive region‑specific analysis of dialysis initiation patterns in a single region. Notably, dialysis initiation rates among older adult patients with DKD or nephrosclerosis gradually increased at the national level, whereas these trends were not significant in Hokkaido. This suggests dialysis initiation among older adults with DKD or nephrosclerosis may have been more limited in Hokkaido, potentially contributing to the overall decline in initiation rates observed in the region. These findings highlight how regional patterns can diverge from national averages, reinforcing the importance of recognizing local heterogeneity when interpreting nationwide statistics.

The 2021/2016 ratios provide a simple and practical measure for assessing recent trends in dialysis initiation rates. These ratios can be readily calculated by members of the JSDT using the WADDA system. Across Japan, the majority of prefectures showed increased ratios ≥ 1 for patients with nephrosclerosis, particularly in older age groups. This trend is likely influenced by Japan’s aging population, as nephrosclerosis, commonly associated with prolonged hypertension, increases in prevalence with advancing age [10, 11]. Despite advances in hypertension treatment, inadequate blood pressure control remains widespread among older adults, highlighting the need for more effective strategies to prevent the progression of nephrosclerosis.

Conversely, dialysis initiation rates among patients with CGN have declined, possibly due to widespread use of renoprotective therapies such as angiotensin-converting enzyme inhibitors, angiotensin receptor blockers [12–14], and sodium-glucose cotransporter-2 inhibitors [15–17]. These medications have been shown to slow the decline in glomerular filtration rate, thereby contributing to reduced dialysis initiation rates. However, despite the overall trend of ratios < 1, regional disparities persist at the prefectural level, with some prefectures exhibiting outlier patterns. Such heterogeneity further supports the notion that uniform national strategies may overlook region-specific challenges and opportunities for intervention. Among patients with DKD aged ≥ 80 years, dialysis initiation rates have been gradually increasing nationwide. This trend highlights the need for earlier detection, such as through participation in routine health checkups, and timely therapeutic intervention. In particular, early initiation of treatment with agents such as angiotensin receptor blockers and sodium-glucose cotransporter-2 inhibitors prescribed by nephrologists may help delay the progression to dialysis in this population [18].

This study has some limitations. First, the study focused exclusively on Hokkaido and did not include comparative analyses with other prefectures that may have exhibited similar or more pronounced trends. Second, the dataset did not allow for sub-prefectural stratification, limiting our ability to assess the impact of geographic factors such as urban–rural disparities or population density. Third, the registry does not capture data on patients who opt for conservative kidney management [19] or decline dialysis, which may contribute to the observed decline in initiation rates. As such, our findings cannot distinguish between true reductions in disease burden and increased dialysis withholding. Fourth, patients aged ≤ 40 years were excluded from the analysis due to the small sample sizes in each subgroup. Fifth, the analysis was limited to the three most common underlying diseases, and comparative analysis with other prefectures was excluded. Finally, while regional differences were observed, the study design does not permit causal inference regarding the underlying drivers of these trends. Although the 2021/2016 ratios provided a simple means of comparison, incorporating additional years and a more detailed geographic breakdown are needed to confirm long-term trends.

In conclusion, dialysis initiation rates in Hokkaido showed a gradual decline across most subgroups, regardless of sex, 10-year age group, or underlying diseases, in contrast to national trends. However, an exception was observed among older adult patients with nephrosclerosis, whose rates increased in parallel with national patterns, highlighting this group as a key target for future intervention strategies. The accessibility and user-friendliness of the WADDA system make it a valuable tool for developing effective, prefecture-specific policies to reduce dialysis initiation throughout Japan.

## Supplementary Information

Below is the link to the electronic supplementary material.

Supplementary file1 (DOCX 691 KB)

Supplementary file2 (XLSX 39 KB)

## Data Availability

The web‑based data system of JSDT is available to all JSDT members at the following address. https://www.jsdt.or.jp/english/.
