# Supplementary material for: Recent trends in dialysis initiation in Japan: a region‑specific descriptive analysis using Hokkaido as an example
Source: Clin Exp Nephrol. 2026 Feb 26;30(4):670–8. doi: 10.1007/s10157-026-02831-y (PMC13009071; doi:10.1007/s10157-026-02831-y)
Supplement: Supplementary file 1 — Supplementary file1 (DOCX 691 KB) [file 10157_2026_2831_MOESM1_ESM.docx]

**Recent trends in dialysis initiation in Hokkaido, northernmost prefecture of Japan**

Hirofumi Sakuma^1^, Megumi Matsumoto^1^, Yusuke Kanno^1^, Saeko Miura^1^, Reina Suetsugu-Ishizawa^1^, Nozomi Hayashi^1^, Motoki Matsuki^1^, Atsushi Wada^2^, and Naoki Nakagawa^1*^

^1^Division of Cardiology and Nephrology, Department of Internal Medicine, Asahikawa Medical University, Asahikawa, Japan

^2^Department of Nephrology, Kitasaito hospital, Asahikawa, Japan

*Corresponding author: Naoki Nakagawa, MD, PhD

Division of Cardiology and Nephrology, Department of Internal Medicine, Asahikawa Medical University, Midorigaoka-higashi 2-1-1-1, Asahikawa, Japan

Phone: +81-166-68-2442, Fax: +81-166-68-2449.

E-mail: [naka-nao@asahikawa-med.ac.jp](mailto:naka-nao@asahikawa-med.ac.jp)

**Supplementary Figure S1.**


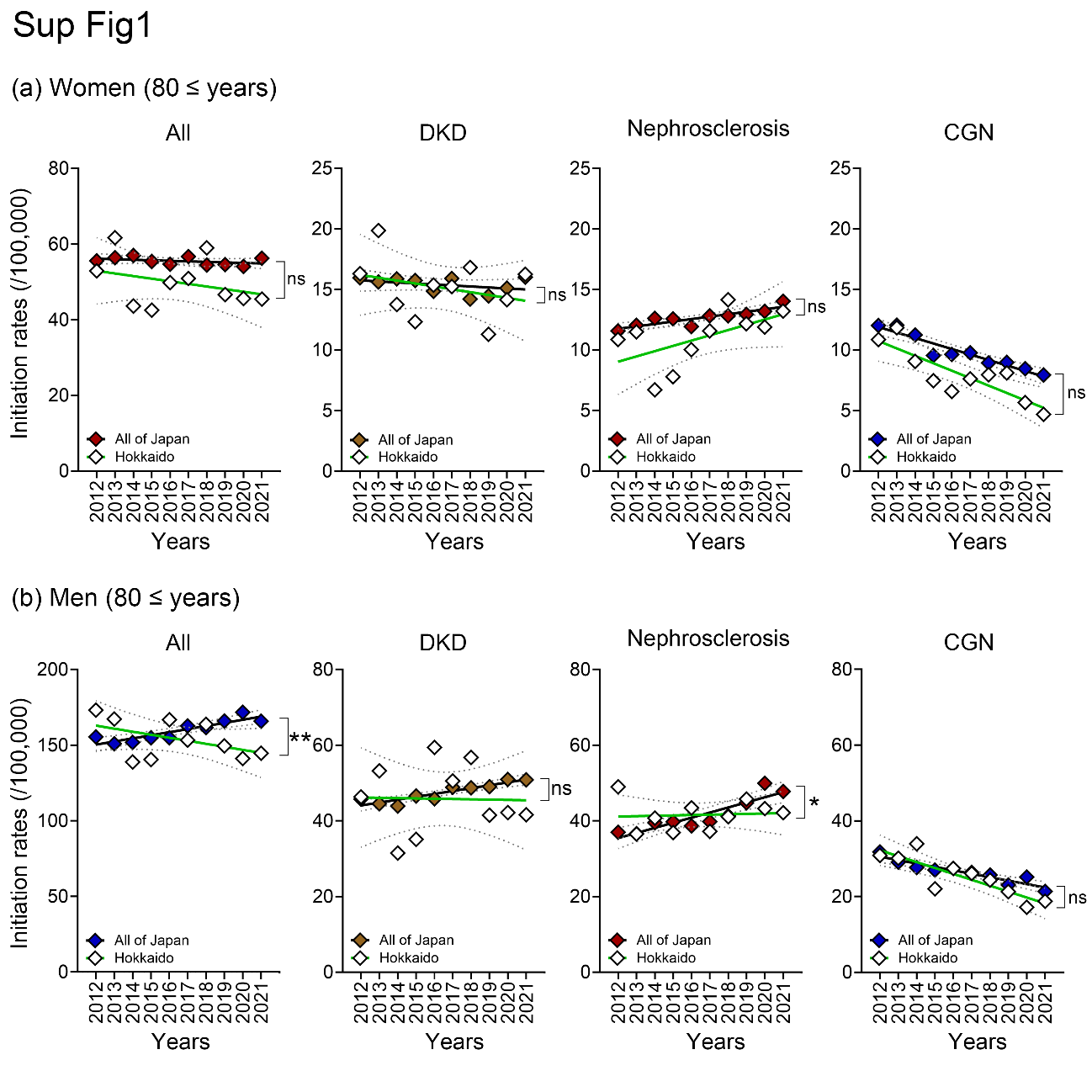


Trends in dialysis initiation rates between 2012 and 2021 among patients aged ≥80 years in each region according to sex and underlying disease. Upper panels represent women (a), and lower panels represent men (b). Each dialysis initiation rate is plotted for all of Japan (filled diamonds) and Hokkaido (open diamonds). Regression lines for all of Japan are shown in black lines and those for Hokkaido are shown in green lines. Regional differences in regression lines were analyzed using an F-test (**p*<0.05, ***p*<0.01 vs. all of Japan). DKD, diabetic kidney disease; CGN, chronic glomerular nephritis.

**Supplementary Figure S2.**


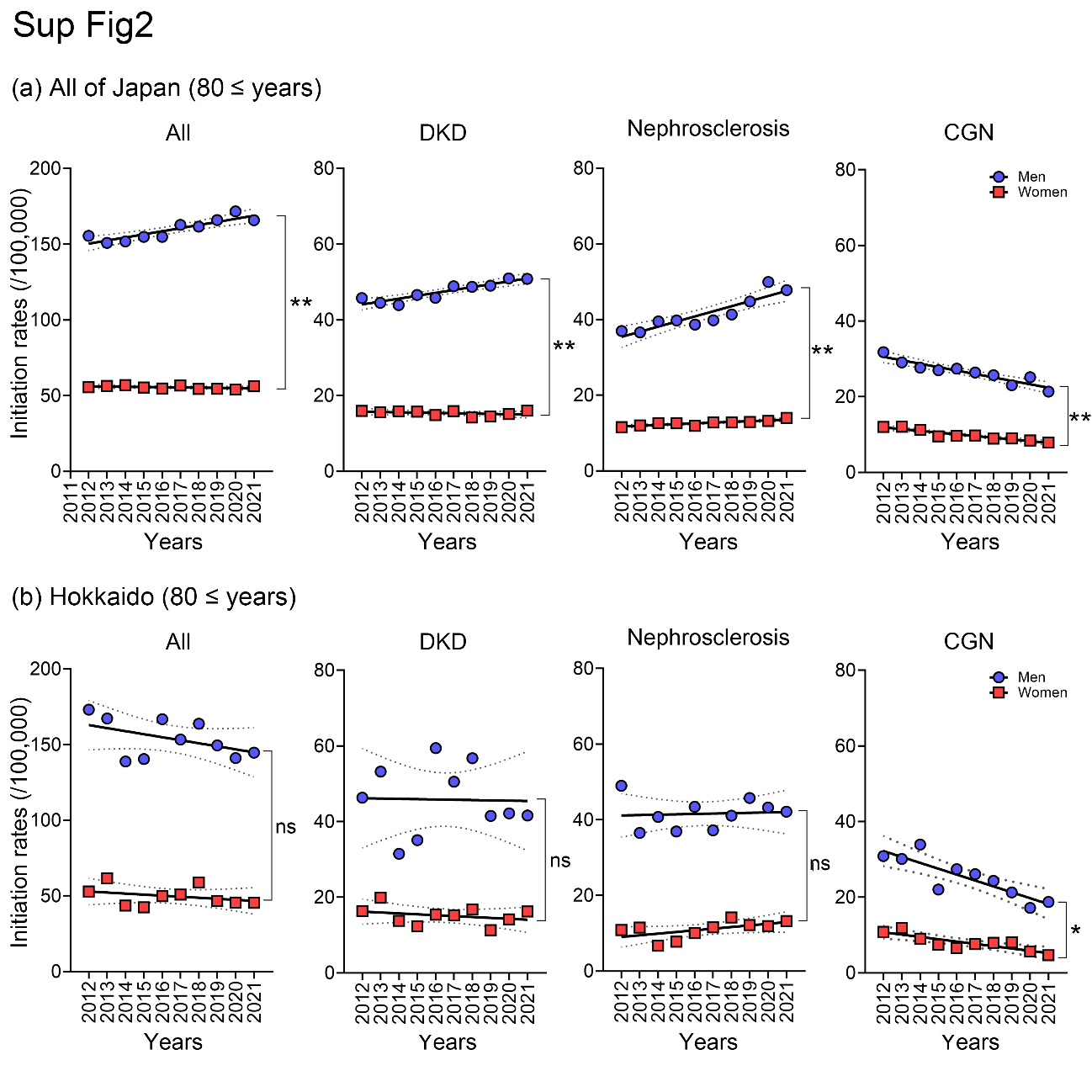


Trends in dialysis initiation rates between 2012 and 2021 among patients aged ≥80 years in each sex according to region and underlying disease. Upper panels represent all of Japan (a), and lower panels represent Hokkaido (b). Each dialysis initiation rate is plotted for women (red squares) and men (blue circles). Regression lines are shown in black lines. Sex differences in regression lines were analyzed using an F-test (**p*<0.05, ***p*<0.01 vs. men). DKD, diabetic kidney disease; CGN, chronic glomerular nephritis.
